# Convalescent or standard plasma versus standard of care in the treatment of COVID-19 patients with respiratory impairment: short and long-term effects. A three-arm randomized controlled clinical trial

**DOI:** 10.1186/s12879-022-07716-5

**Published:** 2022-11-22

**Authors:** Paola Maria Manzini, Giovannino Ciccone, Francesco Giuseppe De Rosa, Rossana Cavallo, Valeria Ghisetti, Sergio D’Antico, Claudia Galassi, Fabio Saccona, Anna Castiglione, Nadia Birocco, Tiziana Francisci, Huijing Hu, Clara Pecoraro, Franca Danielle, Luciana Labanca, Anna Maria Bordiga, Marco Lorenzi, Giovanni Camisasca, Osvaldo Giachino, Mauro Pagliarino, Piero Ottone, Ilvana Tiziana Donatella Scuvera, Roberto Guaschino, Roberto Freilone, Pierluigi Berti, Fabrizia Pittaluga, Maria Avolio, Cristina Costa, Samuele Raso, Aurora Nucci, Massimo Milan, Alessandra Baffa, Alessandra Russo, Antonella Tornello, Laura Maddalena, Grazia Delios, Fabio Paolo Marletto, Anna Grazia De Micheli, Alessio Mattei, Stefano Baldassano, Francesca Canta, Maria Luisa Russo, Daniele Bergamo, Francesco Vitale, Marco Maria Liccardi, Alessandra Chinaglia, Andrea Calcagno, Marcella Converso, Chiara Aldieri, Valentina Libanore, Ilaria Blangetti, Valentina Benedetti, Barbara Mitola, Gitana Scozzari, the PLACO COVID Study Group, Franco Castagno, Adriano Valfrè, Gabriella Rizzioli, Teresa D’Amato, Cristina Crocillà, Silvana Naselli, Valentino Granero, Grazia Cornagliotto, Graziella Lucania, Cristiana Scaglia, Francesca Ferro, Carmela Solimine, Monica Ricotti, Cristina Gilestro, Remigio Roncato, Angela Palladino, Daniela Ongaro, Giulia Anna Poggio, Chiara Chiappero, Simone Mornese Pinna, Silvia Scabini, Federico Vischia, Maria Grazia Gregoretti, Enrico Lupia, Luca Brazzi, Carlo Albera, Luca Scaglione, Valter Gallo, Claudio Norbiato, Roberto Albiani, Bruno Lucio Sini, Andrea Fassiola, Alessandro Locatelli, Giovanni Di Perri, Mauro Navarra, Isabella Gardini, Aurora Ciardiello, Rita La Grotta, Anna De Rosa, Paola Pasquino, Gilberto Fiore, Orietta Franza, Paola Artoni, Stefano Meinardi, Liliana Calosso, Paola Molino, Maria Grazia Veglio, Tiziana Beltramo, Odetta Camerini, Karol Giancaspero, Franca Napoli, Alberto Perboni, Emanuela Messa, Fabrizio Buffolo, Fiammetta Pagnozzi, Stefania Bertone, Lorenzo Lutri, Umberto Gravante, Petros Sacchetti, Alessandra Pavan, Enzo Castenetto, Marco Novelli, Marco Tucciarone, Patrizia Ocello, Giulia Guido, Chiara Frascaroli, Daniela Maria Luisa Vivenza, Francesca Patti, Laura Lorenzelli, Guido Balduzzi, Deborah Ratti, Laura Mazzucco, Valeria Balbo, Francesca Pollis, Sabrina Leoncino, Chiara Lupo, Daniele Romano, Silvia Ziccardi, Melania Marmifero, Guido Chichino, Mario Salio, Giuseppe Aiosa, Riccardo Boverio, Ilaria Avonto, Sara Ghiotto, Riccardo Balbo, Vincenza Nico, Chiara Aguzzi, Maria Chiara Pellegrino, Maristella Prucca, Lucia Assunta Longa, Laura Perotti, Federica Piovano, Luca Ambrogio, Marco Formica, Elisa Monge, Flavia Arena, Nicoletta Barzaghi, Silvia Tavera, Mariaelisa Canepari, Guido Strani, Fulvio Pomero, Maria Grazia Cianci, Mariella Gianarda, Leonardo Ruscitto, Daniel De Martino, Sandro Macchi, Michele Montagnana, Vladimiro Grandinetti, Silvia Magnani, Elisabetta Radin, Valentina Pellu, Monica Meucci, Erika Noè, Paola Torti, Luca Montagnani, Giulio Doveri, Gabriella Giustetto, Costantino Avdis, Marco Prina, Franco Eliantonio, Francesco Lemut, Giuseppe Semino, Palmina Spidalieri, Domenico Vallino, Roberto Prota, Gabriella Buono, Vincenzo Segala, Maria Grazia Milia, Franco Aprà, Sergio Livigni, Emilpaolo Manno, Giuseppe Caula, Emanuela Vitali, Nicola Liuzzi, Mauro Pastorelli, Pietro Caironi, Federica Gamna, Bruno Scapino, Lorenzo Gurioli, Emanuele Magro, Giuseppe Roberti, Gian Mario Santamaria, Antonella Daffonchio, Paola Varese, Gianfranco Ghiazza, Margherita Girino, Carolina Pelazza, Fabrizio Racca, Mirco Grillo, Valerio Del Bono, Giorgio Gianotto, Enzo Aluffi, Enrico Ravera

**Affiliations:** 1 Transfusion Medicine and Blood Establishment, University Hospital City of Science and Health Turin, Corso Bramante 88, 10126 Turin, Italy; 2 Unit of Clinical Epidemiology, University Hospital City of Science and Health Turin, CPO Piemonte, Turin, Italy; 3 Department of Medical Science, University of Turin Faculty of Medicine and Surgery, Turin, Italy; 4 Laboratory of Microbiology and Virology, University Hospital City of Science and Health Turin, Turin, Italy; 5 Laboratory of Microbiology and Virology, Amedeo di Savoia Hospital, Turin, Italy; 6 Oncology Department, University Hospital City of Science and Health Turin, Turin, Italy; 7 Immunohematology and Transfusion Medicine, S Croce and Carle Cuneo Hospital District, Cuneo, Italy; 8 Transfusion Medicine and Blood Establishment, Holy Trinity Hospital Borgomanero, Borgomanero, Italy; 9 Transfusion Medicine, San Giovanni Bosco Hospital, Turin, Italy; 10 Maternal, Pediatric and Trauma Transfusion Medicine, University Hospital City of Science and Health Turin, Turin, Italy; 11 Transfusion Medicine, San Luigi Gonzaga University Hospital, Orbassano, Italy; 12 Immunohematology and Transfusion Medicine, Cardinal Massaia Hospital of Asti, Asti, Italy; 13 Transfusion Medicine, Saints Anthony and Biagio and Cesare Arrigo Alessandria National Hospital, Alessandria, Italy; 14 Transfusion Medicine, Ivrea Hospital, Ivrea, Italy; 15 Immunohematology and Transfusion Medicine, Umberto Parini Hospital, Aosta, Italy; 16 Medical Emergency Division, University Hospital City of Science and Health Turin, Turin, Italy; 17 Pulmunology Unit, University Hospital City of Science and Health Turin, Turin, Italy; 18 Department of Clinical and Biological Science, Faculty of Medicine and Surgery, University of Turin, Turin, Italy; 19 Infectious Diseases Unit, University Hospital City of Science and Health Turin, Turin, Italy; 20 Internal Medicine Unit, Santa Croce Hospital of Moncalieri, Moncalieri, Italy; 21 Internal Medicine Unit, Ordine Mauriziano Di Torino Hospital, Turin, Italy; 22 Intensive Care Unit, Hospital of Chivasso, Chivasso, Italy; 23 Cardiology Unit, Martini Hospital, Turin, Italy; 24 Infectious Diseases Unit, Department of Medical Sciences, University of Turin Faculty of Medicine and Surgery, Turin, Italy; 25 Intensive Care Unit, San Giovanni Bosco Hospital, Turin, Italy; 26 Infectious Diseases, S Croce and Carle Cuneo Hospital District, Cuneo, Italy; 27 Infectious Diseases Unit, Cardinal Massaia Hospital of Asti, Asti, Italy; 28 Intensive Care Unit, Mondovì Hospital, Mondovì, Italy; 29 Internal Medicine Unit, Mondovì Hospital, Mondovì, Italy; 30 Hospital Medical Direction, Ordine Mauriziano di Torino Hospital, Turin, Italy; 31 Molinette Hospital Medical Direction, University Hospital City of Science and Health Turin, Turin, Italy; 32 Internal Medicine 3U, University Hospital City of Science and Health Turin, Turin, Italy; 33 Internal Medicine 5, University Hospital City of Science and Health Turin, Turin, Italy; 34 University Intensive Care Unit, University Hospital City of Science and Health Turin, Turin, Italy; 35 Pulmunology Division, Ordine Mauriziano di Torino Hospital, Turin, Italy; 36 Intensive Care Unit, Maria Vittoria Hospital, Turin, Italy; 37 Intensive Care and Emergency Department, S Croce and Carle Cuneo Hospital District, Cuneo, Italy; 38 Infectious Diseases Unit, Amedeo di Savoia Hospital, Turin, Italy; 39 Intensive Care Unit, Martini Hospital, Turin, Italy; 40 Intensive Care Unit, Santa Croce Hospital of Moncalieri, Moncalieri, Italy; 41 Internal Medicine Department, San Lorenzo Hospital, Carmagnola, Italy; 42 Internal Medicine, Maggiore Hospital, Chieri, Italy; 43 Intensive Care Unit, Maggiore Hospital, Chieri, Italy; 44 Transfusion Medicine, Edoardo Agnelli Hospital, Pinerolo, Italy; 45 Internal and Emergency Medicine, Infermi Rivoli Hospital, Rivoli, Italy; 46 COVID Emergency Medicine, Infermi Rivoli Hospital, Rivoli, Italy; 47 Pulmunology Division, San Luigi Gonzaga University Hospital, Orbassano, Italy; 48 Internal Medicine, Chivasso Hospital, Chivasso, Italy; 49 Internal Medicine, Lanzo Hospital, Lanzo, Italy; 50 Intensive Care Unit, Cardinal Massaia Hospital of Asti, Asti, Italy; 51 Immunohematology and Transfusion Medicine, ASL Alessandria, Alessandria, Italy; 52 Internal Medicine, SS Antonio and Margherita Hospital, Tortona, Italy; 53 Infectious Diseases Unit, Saints Anthony and Biagio and Cesare Arrigo Alessandria National Hospital, Alessandria, Italy; 54 Pulmunology Unit, Saints Anthony and Biagio and Cesare Arrigo Alessandria National Hospital, Alessandria, Italy; 55 Internal Medicine, Saints Anthony and Biagio and Cesare Arrigo Alessandria National Hospital, Alessandria, Italy; 56 Emergency Department, Saints Anthony and Biagio and Cesare Arrigo Alessandria National Hospital, Alessandria, Italy; 57 Medical Department, S Croce and Carle Cuneo Hospital District, Cuneo, Italy; 58 General Medical Department and Rehabilitation, S Croce and Carle Cuneo Hospital District, Cuneo, Italy; 59 Intensive Care and Emergency Department, S Croce and Carle Cuneo Hospital District, Cuneo, Italy; 60 Immonuhematology and Transfusion Medicine, ASL CN1 Savigliano, Savigliano, Italy; 61 Internal Medicine, Michele e Pietro Ferrero Hospital, Verduno, Italy; 62 Immunohematology and Transfusion Medicine, ASL Vercelli, Vercelli, Italy; 63 Immunohematology and Transfusion Medicine, ASL VCO Omegna, Omegna, Italy; 64 Infectious Diseases, Umberto Parini Hospital, Aosta, Italy; 65 Nephrology Unit, Umberto Parini Hospital, Aosta, Italy; 66 Intensive Care Unit, Umberto Parini Hospital, Aosta, Italy; 67 Internal Medicine, Umberto Parini Hospital, Aosta, Italy; 68 Information Technology and Clinical Engineering Unit, University Hospital City of Science and Health Turin, Turin, Italy; 69 Intensive Care Unit, ASL Alessandria, Alessandria, Italy; 70 Transfusion Medicine, SS Antonio and Margherita Hospital, Tortona, Italy; 71 Medical Emergency Division, Ordine Mauriziano di Torino Hospital, Turin, Italy; 72 Semi-intensive Pulmunology Division, Ordine Mauriziano di Torino Hospital, Turin, Italy; 73 Intensive Care Unit, Ordine Mauriziano di Torino Hospital, Turin, Italy; 74 Medical Department, San Giovanni Bosco Hospital, Turin, Italy; 75 Medical Emergency Division, Martini Hospital, Turin, Italy; 76 Internal Medicine, Edoardo Agnelli Hospital, Pinerolo, Italy; 77 Intensive Care Unit, Edoardo Agnelli Hospital, Pinerolo, Italy; 78 Intensive Care Unit, San Luigi Gonzaga University Hospital, Orbassano, Italy; 79 Rehabilitative Unit, San Luigi Gonzaga University Hospital, Orbassano, Italy; 80 Intensive Care Unit, Ivrea Hospital, Ivrea, Italy; 81 COVID Medicine Unit 2, Ivrea Hospital, Ivrea, Italy; 82 Internal Medicine, Ciriè Hospital, Ciriè, Italy; 83 Intensive Care Unit, Ciriè Hospital, Ciriè, Italy; 84 Internal Medicine, SS Antonio and margherita Hospital, Tortona, Italy; 85 Internal Medicine, San Giacomo Hospital, Novi Ligure, Italy; 86 Internal Medicine, Ovada Hospital, Ovada, Italy; 87 Internal medicine, Acqui Terme Hospital, Acqui Terme, Italy; 88 Internal Medicine, Santo Spirito Hospital, Casale M.to, Italy; 89 Research and Innovation Unit, Saints Anthony and Biagio and Cesare Arrigo Alessandria National Hospital, Alessandria, Italy; 90 Intensive Care Unit, Saints Anthony and Biagio and Cesare Arrigo Alessandria National Hospital, Alessandria, Italy; 91 Transfusion Service, Michele e Pietro Ferrero Hospital, Verduno, Italy; 92 Medical Emergency Division, Michele e Pietro Ferrero Hospital, Verduno, Italy; 93 Intensive Care Unit, Michele e Pietro Ferrero Hospital, Verduno, Italy; 94 Immunohematology and Transfusion Medicine, ASL CN1 Mondovì, Mondovì, Italy

**Keywords:** COVID-19 therapy, COVID-19 convalescent plasma, COVID-19 outcomes, Randomized clinical trial

## Abstract

**Background:**

The efficacy of early treatment with convalescent plasma in patients with COVID-19 is debated. Nothing is known about the potential effect of other plasma components other than anti-SARS-CoV-2 antibodies.

**Methods:**

To determine whether convalescent or standard plasma would improve outcomes for adults in early phase of Covid19 respiratory impairment we designed this randomized, three-arms, clinical trial (PLACO COVID) blinded on interventional arms that was conducted from June 2020 to August 2021. It was a multicentric trial at 19 Italian hospitals. We enrolled 180 hospitalized adult patients with COVID-19 pneumonia within 5 days from the onset of respiratory distress. Patients were randomly assigned in a 1:1:1 ratio to standard of care (n = 60) or standard of care + three units of standard plasma (n = 60) or standard of care + three units of high-titre convalescent plasma (n = 60) administered on days 1, 3, 5 after randomization. Primary outcome was 30-days mortality. Secondary outcomes were: incidence of mechanical ventilation or death at day 30, 6-month mortality, proportion of days with mechanical ventilation on total length of hospital stay, IgG anti-SARS-CoV-2 seroconversion, viral clearance from plasma and respiratory tract samples, and variations in Sequential Organ Failure Assessment score. The trial was analysed according to the intention-to-treat principle.

**Results:**

180 patients (133/180 [73.9%] males, mean age 66.6 years [IQR 57–73]) were enrolled a median of 8 days from onset of symptoms. At enrollment, 88.9% of patients showed moderate/severe respiratory failure. 30-days mortality was 20% in Control arm, 23% in Convalescent (risk ratio [RR] 1.13; 95% confidence interval [CI], 0.61–2.13, P = 0.694) and 25% in Standard plasma (RR 1.23; 95%CI, 0.63–2.37, P = 0.544). Time to viral clearance from respiratory tract was 21 days for Convalescent, 28 for Standard plasma and 23 in Control arm but differences were not statistically significant. No differences for other secondary endpoints were seen in the three arms. Serious adverse events were reported in 1.7%, 3.3% and 5% of patients in Control, Standard and Convalescent plasma arms respectively.

**Conclusions:**

Neither high-titer Convalescent nor Standard plasma improve outcomes of COVID-19 patients with acute respiratory failure.

*Trial Registration* Clinicaltrials.gov Identifier: NCT04428021. First posted: 11/06/2020

**Supplementary Information:**

The online version contains supplementary material available at 10.1186/s12879-022-07716-5.

## Background

Given the lack of evidence for effective treatment of COVID-19 during the first wave of pandemic, empirical and historical interventions have re-emerged as options for the control of the disease. That is the case of convalescent plasma, which has been considered an emergency intervention in several pandemics [1–5]. Initially available observational or control matched studies on COVID-19 patients were encouraging, suggesting that COVID-19 Convalescent Plasma (CCP) could reduce mortality, improve clinical outcomes, and confirming its safety [6–13]. The majority of those studies suggested that treatment in early phases of infection and high titer antibodies could represent the keys for its efficacy. However, at the time the current trial was designed, it had not been investigated whether the potential efficacy of CCP could be attributable only to its specific antibody content or if other substances in plasma, as anti-inflammatory cytokines and natural or acquired antibodies, could exert positive immunomodulation effects. Thus, due to tolerability and potential benefits at that time, we designed a 3-arms randomized trial to explore the effectiveness of high titer CCP or Standard Plasma (SP) in early phases of infection as therapeutic options to add to Standard of Care (SC) to control short and long-term progression of the disease.

Preliminary results on 14-days mortality of control and COVID-19 Convalescent Plasma arms have been included in an international metanalysis on published, unpublished and ongoing randomized trials all over the world [14].

## Methods

This study was a randomized, three-arms, blinded on interventional arms, multicentric trial conducted at 19 hospitals (listed in the Study Protocol in Additional file 1) in Piedmont and Valle d’Aosta Regions (North-Western Italy).

An independent Data and Safety Monitoring Committee was settled to verify study protocol, trial conduction and perform an interim analysis to assess safety and efficacy at 40% of enrolment. The authors take full responsibility for the design, conduct, and analysis of the trial in adherence to the study protocol and guarantee the accuracy and completeness of the data.

Hospitalized adults (age > 18 yrs) with a reverse-transcriptase–polymerase-chain-reaction (RT-PCR) confirmed SARS-CoV-2 infection on a nasopharyngeal swab or bronchoalveolar lavage, and a radiologically confirmed pneumonia with a respiratory impairment onset within five days were eligible for enrollment.

Exclusion criteria were: pregnancy, previous severe reactions to plasma infusion, and unavailability of AB0 compatible CCP.

**Fig. 1:**
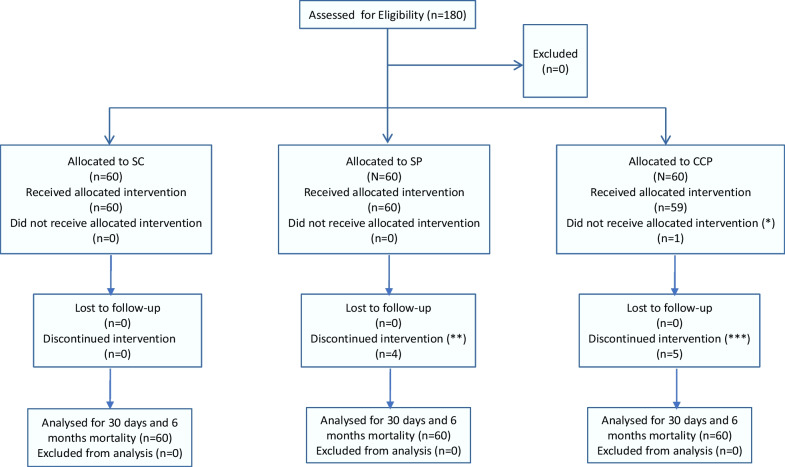
Study flow with patients enrolled, randomized and analyzed for primary endpoint. SC: Standard of Care therapy (control arm). SP: Standard Plasma (experimental arm). CCP: Covid-19 Convalescent Plasma (experimental arm). *The patient withdrew the consent the day after randomization before starting treatment. **Four patients died after second SP infusion. ***2 patients died after first and 2 after second CCP infusion, 1 patient withdrew the consent after second infusion because of a moderate allergic reaction

After assessing eligibility and availability of AB0 compatible CCP, treating physicians informed hospitalized patients about the trial protocol and asked to sign a written informed consent. Those who accepted, after entering the baseline data on EPICLIN (https://new.epiclin.it/it/placo/), a website-based platform, were automatically stratified by severity of respiratory impairment in three groups:

- mild: partial pressure of oxygen (PaO2) ≥ 60 mmHg in ambient air (aa) with non-invasive supplemental oxygen
- moderate: PaO2 < 60 mmHg in aa in non-invasive ventilation (NIV) or in Continuous Positive Airway Pressure (CPAP)
- severe: suspected or confirmed acute respiratory distress syndrome (ARDS) in CPAP or mechanical ventilation (MV) ± Extra Corporeal Membrane Oxygenation (ECMO). ARDS (according to Berlin definition) was suspected when a rapid reduction of PaO2/FIO2 towards 300 mmHg was observed.

and then randomized in a 1:1:1 ratio according to a computerized generated sequence (for details on randomization see the Study Protocol available in the online version—see Supplementary Information). Study flow is presented in Fig. 1.

The trial used a blinded interventional arm design. The web-based random procedure was unpredictable by all those involved in the study, who received only the assignment either to the standard arm or to the experimental arms with plasma. Only the transfusion Centers knew the type of plasma (SP or CCP) assigned to patients in the experimental arms. They were responsible for blinding the three plasma bags (SP or CCP) and accompanying certificates, with black tags reporting “TRIAL PLASMA”.

Participants were randomized to receive either Standard of care or SC + three units of Standard Plasma (SP) collected in the pre-COVID-19 era (before September 2019), or SC + three units of high-titre CCP.

The SC was not strictly defined, but the trial protocol recommended to follow national or international updated guidelines for COVID-19. CCP was collected in May and June 2020 from donors recovered from first-wave COVID-19 infection, when the predominant variant in our area was the 20A S614G lineage (https://clades.nextstrain.org/). The plasma units were administered on days 1, 3, and 5 after randomization. Plasma infusion was discontinued whether a severe life-threatening reaction to transfusion happened or in case of withdrawal of written consent for any reason.

97% of the units used in the trial had IgG anti-SARS-CoV-2 > 40 Arbitrary Units (AU)/ml by a quantitative Chemiluminescence-Immunoassay (CLIA) (LIAISON^®^ SARS-CoV-2 S1/S2 IgG) showing to be concordant with Plaque Reduction Neutralization Test (PRNT): 40AU/ml = PRNT titer > 1:80. The three doses (100–300 ml each) of CCP were chosen possibly from different donors to reach a total median administered amount of 70.000 AU of neutralizing antibodies.

A detailed description of COVID-19 convalescent donors and of CCP process methods are presented in Additional file 1.

The primary outcome was 30-days mortality rate.

Secondary outcomes were: incidence of mechanical ventilation (MV) or death at day 30, 6-month mortality rate, proportion of days with MV (originally defined as days in ICU) on total length of hospital stay, proportion of patients showing seroconversion to IgG anti-SARS-CoV-2, viral clearance by RT-PCR on plasma and respiratory tract samples, and variations in Sequential Organ Failure Assessment (SOFA) score from randomization, with assessments on day 2, 4, 6, 10, 14, 21, 28 or until discharge or death. Laboratory methods of SARS-CoV-2 RNA extraction and quantitation, and SOFA score are described in Additional file 1.

Complete data were not available for the other endpoint defined in the protocol (proportion of patients with drug treatment modification).

Safety outcome was the proportion of patients developing any Adverse Events (AEs) assessed daily from randomization to day 30 or discharge or death. Definition of AEs is reported in Additional file 1, as well as complete participant timeline for blood tests and clinical parameters to be reported on daily Case Report Forms.

The study design involves comparing each of the two experimental arms with the control without correction for multiplicity [15]. We calculated a sample size of 180 patients (58 per arm, rounded to 60) to assess a reduction from 25 to 10% of 30-days mortality (primary endpoint), with an alpha error (1-tail) of 0.10 and a statistical power of 80%.

The trial was analysed according to the intention-to-treat principle.

The comparisons of the proportion of deaths and of MV or death at 30 days and proportion of deaths at 6 months were stratified (by the severity of respiratory failure) and estimated in terms of RR with a Mantel–Haenszel Chi-square test. A secondary analysis, adjusted for the stratification criterion and few unbalanced critical prognostic factors (age, sex, BMI, CCI, blood group), was conducted with a Poisson regression model to estimate adjusted RRs. Subgroup analyses for the primary endpoint were performed, including in the same model the interaction terms between treatment arms and the following variables: (a) planned: stratification level, age group (< 65; 65–74; 75 + years), sex, and (b) exploratory: blood group (A *vs* others), days from symptom onset to randomization (0–5, 6–10, ≥ 11) and the viraemic and serologic test results (positive, negative) at baseline. Cumulative incidence of virus clearance from plasma and respiratory tract samples and seroconversion to IgG anti-SARS-CoV-2 were compared in terms of sub-distribution Hazard Ratios (sHR) with Fine and Gray models, considering death or discharge as competing events.

We compared the percentage of MV days using an ordinal logistic model and variations of serum IgG anti-SARS-CoV-2 levels and SOFA scores during hospitalization using generalized linear mixed models for repeated measures. Statistical analyses were performed with SAS v. 9.4 and STATA v.15.

## Results

From June 2020 to February 2021, 180 patients (73.9% males) were enrolled in the trial, the majority between October 2020 and January 2021, during the second pandemic wave; follow-up ended in December 2021. Demographic, clinical characteristics and treatment at baseline of the enrolled patients are listed in Table 1. The median age was 66.6 years (IQR 57.0–73.0). Most patients (88.9%) showed moderate to severe respiratory failure at enrollment, with a mean SOFA score of 2.99 (SD 1.66).

**Table 1:** Demographic and clinical characteristics of patients and standard drug therapy at baseline

	Standard of Care (SC) (N = 60)		SC + Standard Plasma (N = 60)		SC + COVID-19 Convalescent Plasma (N = 60)		Total (N = 180)	
Sex—no (%)								
Males	40	66.7	47	78.3	46	76.7	133	73.9
Females	20	33.3	13	21.7	14	23.3	47	26.1
Age (years)—no (%)								
< 55	11	18.3	13	21.7	11	18.3	35	19.4
55–64	14	23.3	12	20.0	18	30.0	44	24.4
65–74	24	40.0	20	33.3	21	35.0	65	36.1
75 +	11	18.3	15	25.0	10	16.7	36	20.0
Median—(IQR)	67.5 (58.5–72.0)		67.0 (56.5–74.5)		65.0 (57.5–73.0)		66.6 (57.0–73.0)	
Charlson Comorbidity Index—no (%)								
0	34	56.7	36	60.0	31	51.7	101	56.1
1	12	20.0	10	16.7	19	31.7	41	22.8
2–3	8	13.3	9	15.0	9	15.0	26	14.4
4 +	6	10.0	5	8.3	1	1.7	12	6.7
Comorbidities—no (%)								
Cardiovascular diseases	9	15.0	10	16.7	14	23.3	33	18.3
Cerebrovascular diseases	3	5.0	1	1.7	1	1.7	5	2.8
Chronic pulmonary diseases	3	5.0	8	13.3	3	5.0	14	7.8
Diabetes	9	15.0	11	18.3	9	15.0	29	16.1
Chronic kidney diseases	1	1.7	4	6.7	1	1.7	6	3.3
Liver diseases	2	3.3	1	1.7	1	1.7	4	2.2
Previous neoplasia	7	11.7	5	8.3	6	10.0	18	10.0
Hypertension	25	41.7	24	40.0	20	33.3	69	38.3
Solid organ transplant	1	1.7	1	1.7	2	3.3	4	2.2
Body Mass Index—no (%)								
< 25	19	31.7	15	25.0	14	23.3	48	26.7
25–29	27	45.0	27	45.0	29	48.3	83	46.1
30 +	10	16.7	14	23.3	16	26.7	40	22.2
n.d	4	6.7	4	6.7	1	1.7	9	5.0
Blood group—no (%)								
N/A	4	6.7					4	2.2
0	27	45.0	28	46.7	26	43.3	81	45.0
A	23	38.3	25	41.7	30	50.0	78	43.3
AB	1	1.7	2	3.3	1	1.7	4	2.2
B	5	8.3	5	8.3	3	5.0	13	7.2
Onset of symptoms—days								
0–5	16	26.7	16	26.7	15	25.0	47	26.1
6–10	21	35.0	23	38.3	25	41.7	69	38.3
11 +	22	36.7	20	33.3	20	33.3	62	34.4
Median (IQR)—days	9 (5–12)		8 (5–12)		8 (6–11,5)		8 (5–12)	
Symptoms at onset—no (%)								
Fever	52	86.7	46	76.7	48	80.0	146	81.1
Cough	35	58.3	31	51.7	33	55.0	99	55.0
Exertional Dyspnea	34	56.7	29	48.3	28	46.7	91	50.6
Nausea/ Diarrhea	9	15.0	7	11.7	6	10.0	22	12.2
Fatigue	9	15.0	12	20.0	12	20.0	33	18.3
Myalgia	7	11.7	8	13.3	10	16.7	25	13.9
Anosmia/Ageusia	4	6.7	7	11.7	7	11.7	18	10.0
Others	7	11.7	5	8.3	2	3.3	14	7.8
Onset of respiratory failure—days								
0–1	21	35.0	19	31.7	21	35.0	61	33.9
2–3	28	46.7	31	51.7	28	46.7	87	48.3
4–5	11	18.3	10	16.7	11	18.3	32	17.8
Median (IQR)—days	2 (1–3)		2 (1–3)		2 (1–3)		2 (1–3)	
Degree of respiratory failure—no (%)								
Mild	7	11.7	6	10.0	7	11.7	20	11.1
Moderate-severe	32	53.3	32	53.3	31	51.7	95	52.8
Severe	21	35.0	22	36.7	22	36.7	65	36.1
Oxygen supplementation devices—no (%)								
Low flow nasal cannula	2	3.3	6	10.0	9	15.0	17	9.4
Venturi Mask ± reservoir	12	20.0	7	11.7	8	13.3	27	15.0
High flow nasal cannula	4	6.7	3	5.0	1	1.7	8	4.4
NIV/CPAP	38	63.3	44	73.3	41	68.3	123	68.3
Mechanical Ventilation	4	6.7			1	1.7	5	2.8
SOFA score—mean (SD)	3.1 (1.80)		2.96 (1.43)		2.9 (1.74)		2.99 (1.66)	
Plasma SARS-CoV-2 RNA—no (%)								
Negative	18	30.0	19	31.7	16	26.7	53	29.44
Positive	42	70.0	41	68.3	44	73.3	127	70.6
Plasma IgG anti SARS-CoV-2—no (%)								
Negative	20	33.3	19	31.7	29	48.3	68	37.8
Positive	40	66.7	41	68.3	31	51.7	112	62.2
Median IgG anti-SARS-CoV-2—median (IQR)	40.1 (6.1–89.8)		20.4 (9.7–40.9)		15.8 (4.4–53)		22.6 (6.0–67.5)	
Treatments at enrolment—no (%)								
Heparin	57	95.0	56	93.3	54	90	167	92.8
Glucocorticoids	56	93.3	59	98.3	54	90	169	93.9
Antibiotics	43	71.7	41	68.3	45	75	129	71.7
Remdesivir	13	21.7	8	13.3	9	15	30	16.7
Tocilizumab	1	1.7	2	3.3	3	5	6	3.3
Other Immunosuppressants	2	3.3	1	1.7	3	5	6	3.3
Combinations								
Heparin + glucocorticoids + antibiotics	41	68.3	39	65.0	38	63.3	118	65.6
Heparin + glucocorticoids	13	21.7	17	28.3	12	20.0	42	23.3
Glucocorticoids + antibiotics or other drugs	1	1.6	2	3.3	2	3.3	6	3.30

IQR denotes Interquartile range. NIV indicates non-invasive ventilation, CPAP Continuous Positive Airway Pressure, SARS-CoV-2 severe acute respiratory syndrome coronavirus 2, and SOFA Sequential Organ Failure Assessment

The three arms were well balanced for COVID-19 related variables, with some unbalances for age, sex, BMI and blood groups.

The mean amount of IgG anti-SARS-CoV-2 administered to a single patient with three doses of CCP was 93,431 AU, comparable to three 350 ml units with a PRNT > 1:160. 56/60 patients (93.3%) in the SP arm and 54/60 (90%) in the CCP arm completed plasma infusion. Eight patients (four in each experimental arm) died within day 5 (2 deaths after the first infusion in CCP arm and 6 deaths after the second infusion: 4 in the SP arm and 2 in CCP arm). Furthermore, in the CCP arm, two patients withdrew consent for infusion, one, the day after enrollment, before the first infusion and one after the second infusion, because of a moderate allergic reaction.

Overall, with 41 deaths out of 180, the overall 30-days mortality rate was 22.8% (95%CI: 17.3–29.4), with increasing risks according to the severity of respiratory failure at enrollment (mild: 10%, intermediate: 13.7% and severe: 40%).

**Fig. 2:**
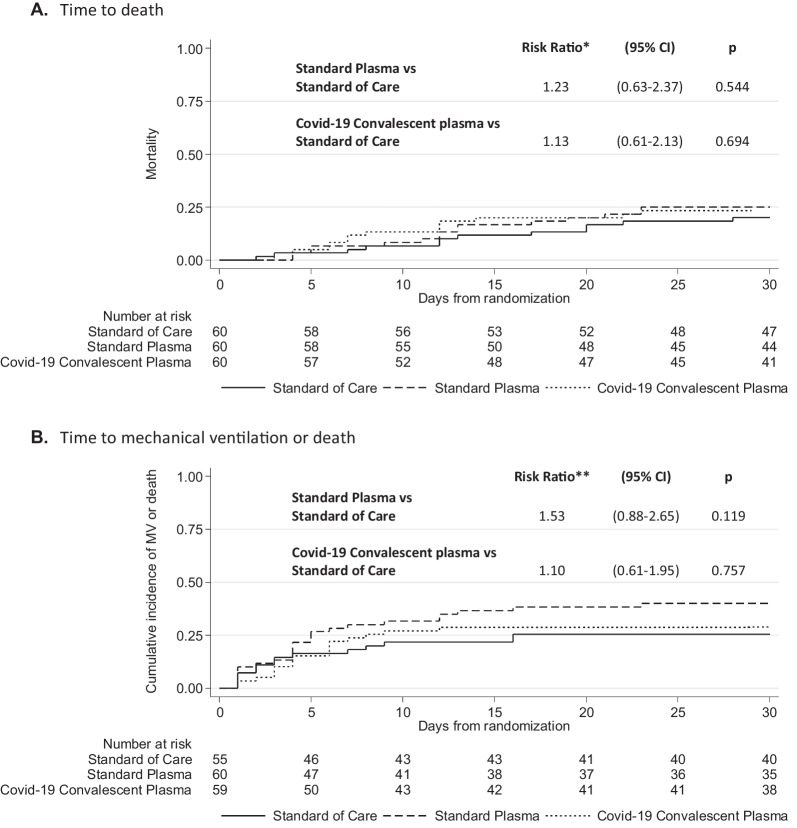
Cumulative incidence of death (**A**) and mechanical ventilation or death (**B**) by treatment arm. *Risk ratio of 30-day mortality, stratified by severity of respiratory impairment. **Risk ratio of 30-day mechanical ventilation or death, stratified by severity of respiratory impairment

**Fig. 3:**
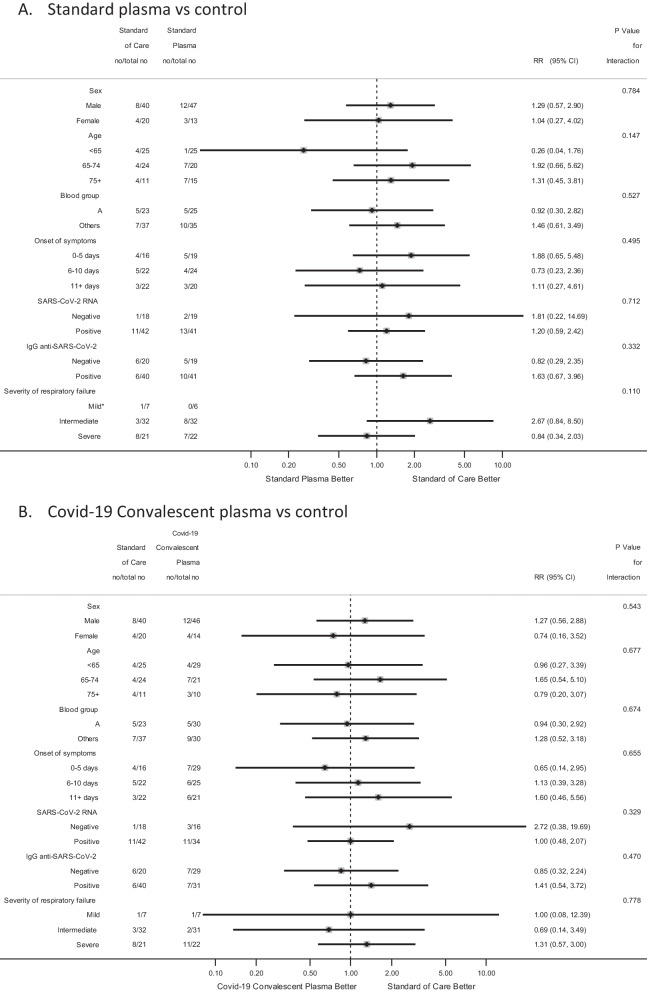
Forest plot with subgroup comparisons of Standard plasma vs Control (**A**) and COVID-19 Convalescent plasma vs Control (**B**). *Not estimated because no events were observed in Standard Plasma arm

In comparison with patients treated with SC, who experienced a 30-days mortality of 20%, no reductions were seen for patients treated with CCP (23.3%; RR 1.13; 95%CI, 0.61–2.13, P = 0.694) or with SP (25.0%; RR 1.23; 95%CI, 0.63–2.37, P = 0.544) (Fig. 2A and Table 2). These results were confirmed with a multivariable model including age, sex, BMI, CCI, blood group, and the stratification variable (severity of respiratory failure) (Additional file 1: Table 1s) as well as by subgroup analyses (Fig. 3).

**Table 2:** Study endpoints and comparisons between the experimental arms and control arm in the intention to treat population

	Control arm		Experimental arms				Standard plasma *vs* control			COVID-19 Convalescent Plasma *vs* control		
	Standard of Care (N = 60)		Standard Plasma (N = 60)		COVID-19 Convalescent Plasma (N = 60)							
	no./total no	(%)	no./total no	(%)	no./total no	(%)	Risk Ratio	(95% CI)	p	Risk Ratio	(95% CI)	p
Primary endpoint												
30-days mortality	12/60	(20.0)	15/60	(25.0)	14/60	(23.3)	1.23	(0.63–2.37)	0.544	1.13	(0.61–2.13)	0.694
Secondary endpoints												
30 days-incidence of Mechanical Ventilation or death	14/56	(25.0)	24/60	(40.0)	17/59	(28.8)	1.53	(0.88–2.65)	0.119	1.10	(0.61–1.95)	0.757
6-months mortality	16/60	(26.7)	16/60	(26.7)	14/60	(23.3)	0.98	(0.55–1.76)	0.951	0.85	(0.48–1.53)	0.600

							Sub Hazard Ratio	(95% CI)	p	Sub Hazard Ratio	(95% CI)	p
Time (days) to seroconversion to IgG anti-SARS-CoV-2*	3	(2–4)	2	(2–4)	2	(2–4)	1.38	(0.79–2.39)	0.255	1.54	(0.89–2.66)	0.119
Time (days) to RT-PCR viral clearance on plasma: median (IQR)**	5	(4–6)	6	(4–6)	6	(4–6)	1.01	(0.67–1.53)	0.957	0.94	(0.62–1.42)	0.761
Time (days) to RT-PCR viral clearance on respiratory tract samples: median (IQR)	23	(21–28)	28	(20–37)	21	(15–22)	0.99	(0.56–1.77)	0.983	1.24	(0.71–2.17)	0.457

							OR***	(95% CI)	p	OR***	(95% CI)	p
Percentage of Mechanical Ventilation days: mean (SD)	11.58	(26.45)	10.37	(22.23)	8.81	(22.70)	1.03	[0.42, 2.53]	0.950	0.79	[0.30, 2.08]	0.638

							Mean difference	(95% CI)	p	Mean difference	(95% CI)	p
SOFA score variations during hospitalization: mean (SD) ****	0.89	(3.61)	0.61	(2.83)	0.11	(3.38)	− 0.34	(− 1.16, 0.48)	0.419	− 0.70	(− 1.57, 0.15)	0.107
IgG anti-SARS-CoV-2 variations during hospitalization: mean (SD) ****	88.54	(82.61)	97.66	(96.59)	80.19	(64.12)	− 2.12	(− 24.99, 20.76)	0.856	− 11.47	(− 32.97, 10.03)	0.296

IQR denotes Interquartile range, LOS length of hospital stay, and RT-PCR reverse-transcriptase–polymerase-chain-reaction

*N = 68 patients without anti-SARS-CoV-2 IgG at baseline (20 Standard of Care, 19 Standard Plasma, 29 COVID-19 Convalescent Plasma). **N = 125 patients with positive RT-PCR viral RNA at baseline (41 Standard of Care, 41 Standard Plasma, 43 COVID-19 Convalescent Plasma). ***OR for an increase of 10% of Percentage of Mechanical Ventilation days ****Reported mean (SD) at day 10

Incidence of the composite endpoint of MV or death within 30 days was not improved in the experimental arms compared to SC (Fig. 2B and Table 2).

At 6 months, with 46 deaths out of 180, the overall mortality rate was 25.6% (95%CI 19.7–32.4). In comparison with patients treated with SC, no clear differences were seen for patients treated with SP (RR 0.98; 95%CI, 0.55–1.76, P = 0.951) or with CCP (RR 0.85; 95%CI, 0.48–1.53, P = 0.600).

Sixty-eight patients (38%) had undetectable IgG anti SARS-CoV-2 at enrollment, 51 patients (28%) had an antibody titer lower than that of CCP units (< 40 AU/ml), while 61 (34%) had an antibody titer > 40 AU/ml. Time to seroconversion was slightly shorter for CCP (sHR 1.54) and SP (sHR 1.38), but the differences between medians were minor (1 day) and statistically weak (Additional file 1: Figure 1sA). 127 patients (70.6%) showed the presence of SARS-CoV-2-RNA by RT-PCR in plasma samples at baseline and other 17 during follow-up. Viremia became negative in a median time of 5 or 6 days (IQR 4–6), without differences between arms (Additional file 1: Figure 1sB).

Median time to viral clearance in the respiratory tract was reached in a slightly shorter time in the CCP arm (21 days) than in SC (23 days), but the difference was not statistically sound (Additional file 1: Figure 1sC).

The median length of hospital stay for the entire population was 15 days, slightly shorter in patients who received CCP or SP than SC (14 and 15.5 vs 17.5, respectively).

The proportion of days of MV on the total length of hospital stay was 10.2%, without meaningful differences between the three arms. A mean reduction from baseline of − 0.70 (95% CI, 1.57–0.15, P = 0.107) of the SOFA score during hospitalization was recorded in patients in the CCP arm compared to the SC arm. In contrast, no apparent differences in IgG seroconversions between arms were recorded (Additional file 1: Figure 2sA and B).

A descriptive analysis of the frequency and percentage of patients with altered laboratory values at baseline and during hospitalization (within 30 days since randomization) and 30-day mortality, by treatment arm, is reported in Additional file 1: Table 2s. As expected, for all the variables analysed, especially for D-Dimer and CRP, a strong positive association between altered values and increased mortality was evident.

Details of AEs are described in the Additional file 1: Table 3s. We observed 4 AEs to plasma infusion, 2 in each arm. Severe AEs were: 3 pulmonary thromboembolism, 1 massive cerebral hemorrhage during ECMO, 1 myocardial infarction, 1 iatrogenic pneumothorax (reported in 1.7%, 3.3% and 5% of patients in Control, SP and CCP arms respectively) and 41 deaths (for respiratory failure in all cases).

## Discussion

At variance with most previous non randomized studies, but in accordance with nearly all randomized controlled trials, we failed to demonstrate any significant improvement of relevant clinical outcomes adding COVID-19 convalescent plasma to the standard of care even though we tried to anticipate as much as possible the treatment and to use high doses of antibodies [10, 16–28]. A trend to a shorter length of hospital stays and a reduction in MV incidence and duration through the hospital stay was seen for CCP treatment, but differences were small and statistically weak. Moreover, no improvement was seen in outcomes by adding SP to SC, thus proving that anti-inflammatory cytokines and natural or acquired antibodies contained in standard plasma did not help in this phase of COVID-19 disease.

Our inclusion criteria permitted enrollment within 5 days since onset of respiratory impairment that was the shortest possible interval considering standards for inpatients in our hospitals (patients were advised to come to the hospital only if respiratory impairment was present). This led to a median interval of 8 days (range 5–12) since the onset of symptoms. Clinical results of our trial are in accordance with those of most randomized controlled studies published to date [14, 17–19, 24, 26, 27, 29, 30], that showed no efficacy of CCP for patients with comparable time since onset of symptoms. Different results, with a reduced risk of evolution of the disease, have only been shown in a single randomized trial [25] that used CCP within 3 days since onset of symptoms, before pneumonia and its complications became clinically evident at variance with other two more recent papers that failed to confirm the efficacy of early use [22, 31]. Our subgroup analyses only suggest a decreasing effect of CCP with increasing time from symptom onset, but the evidence is weak.

In contrast to a randomized trial that was interrupted because 79% of patients at enrollment were showing comparable antibodies titers than CCP [18], in our study only 34% of patients had antibodies titers higher than the lower CCP antibody titers (40 AU/ml) so most patients being in a very early phase of infection before an immune response was appreciable. Nevertheless, even in this early phase, when antibodies titers are not yet raised, passive immunotherapy didn’t seem to play a crucial role in shortening disease history, preventing complication or ameliorating clinical outcomes.

It has also been suggested that the titer of neutralizing antibodies plays a crucial role in the effectiveness of CCP treatment [28]. Our trial neutralizing antibodies total dose, even though no precise comparison amongst trials can be made to date, can be considered one of the highest administered in a randomized trial so far. We enlarged the total volume of CCP administered to patients compared to other studies (3 units). All our plasma units were tested with an ELISA assay that correlates with PRNT, and we estimated a mean infusion of three 350 ml units with a PRNT > 1:160. All participants received plasma from at least two donors (15 patients from 3 donors) trying to increase antibodies heterogenicity. Furthermore, what differs from other treatments is the attempt to standardize the total amount of antibodies administered per patient. A mean total dose of 93,000 AU of antibodies was administered to patients in CCP arm. Nevertheless, no advantage in outcomes was seen with this high dose strategy compared to SC in this phase of the disease. In light of suggestions from a recent paper [29] showing that patients treated with high levels of anti-Spike protein CCP showed worse outcomes, the ELISA test we used, detecting anti-Spike-protein antibodies, despite the excellent correlation with PRNT, could have selected CCP with unfavorable antibody profile for COVID-19 patients’ treatment.

In our study, most enrolled patients (88.9%) were affected by moderate to severe respiratory failure. This selection reflects the greater propensity of physicians to propose study participation to most severe than to mild cases that could be considered a limit of our study. The high prevalence of patients (80%) with detectable SARS-CoV-2 viremia, one of the highest described in the literature, confirms the severity of the disease in our cohort of patients. Our results confirm previous studies showing worse outcomes and increased mortality in plasma RNA + patients, irrespective of treatment [32–36]. Furthermore, CCP did not increase the clearance of SARS-CoV-2 viremia from plasma, indicating that passive immunization does not play a key role for infection in this phase of the disease.

A slightly faster clearance of virus from respiratory tract samples was seen in CCP patients in accordance with other data [6, 7, 16, 18]. Still, this difference was not statistically sound and is of questionable clinical relevance.

On the other side, no meaningful difference was seen in our trial in number or types of AEs between three arms of treatment confirming the safety of SP and CCP in this subset of patients [37, 38]. The three cases of thromboembolism observed in our trial, one in each treatment arm, were not related to plasma infusion. With strict daily monitoring of possible AEs, our trial confirms that this amount of plasma (mean = 230 ml, equivalent to 3 ml per Kg) is neutral in terms of coagulation processes in vivo, probably providing a balanced amount of procoagulants and anticoagulant factors.

The inclusion of a study arm with SP, the careful selection of CCP units to administer a comparable dose of antibodies to all treated patients, the masking of the plasma bags, the evaluation of SARS-CoV-2-RNA on patients plasma and in the respiratory tract over time, the strict monitoring of clinical data and the 6 months follow-up represent the originality and the strengths of our study. Due to the substantial expected benefits, the relatively small sample size is the major limitation of our study.

## Conclusions

Our study supports the findings from almost all randomized controlled trials that CCP does not offer meaningful therapeutical advantages over standard care in fighting against COVID-19 disease and its complications after the onset of respiratory failure and confirms that there is no reason to continue to use CCP in this subset of patients. Furthermore, it underlines that SP and its potential immune-modulatory effect has no impact on this clinical condition.

## Supplementary Information

**Additional file 1.** Supplementary appendix.

## Data Availability

The datasets used and/or analysed during the current study are not publicly available due to database complexity for structure and amount of collected data, that request a precise and clear definition of dataset requested and precise objectives before sharing. Data are in any case available from the corresponding author on reasonable request.

## Abbreviations

CCP: COVID-19 convalescent plasma
SP: Standard plasma
SC: Standard of care
RT-PCR: Reverse-transcriptase–polymerase-chain-reaction
PaO2: Partial pressure of oxygen
AA: Ambient air
NIV: Non-invasive ventilation
CPAP: Continuous Positive Airway Pressure
ARDS: Acute respiratory distress syndrome
MV: Mechanical ventilation
ECMO: Extra Corporeal Membrane Oxygenation
AU: Arbitrary units
CLIA: Chemiluminescence-Immunoassay
PRNT: Plaque Reduction Neutralization Test
SOFA score: Sequential Organ Failure Assessment score
AEs: Adverse events
sHR: Sub-distribution Hazard Ratios
