# Supplementary material for: Convalescent or standard plasma versus standard of care in the treatment of COVID-19 patients with respiratory impairment: short and long-term effects. A three-arm randomized controlled clinical trial
Source: BMC Infect Dis. 2022 Nov 22;22:879. doi: 10.1186/s12879-022-07716-5 (PMC9682750; doi:10.1186/s12879-022-07716-5)
Supplement: Supplementary file 1 — Additional file 1. Supplementary appendix. [file 12879_2022_7716_MOESM1_ESM.docx]

Supplementary appendix

Manzini P. et al. **Convalescent or Standard plasma versus Standard of care in the treatment of Covid-19 patients with respiratory impairment: short and and long-term effects.**

**A three-arm randomized controlled clinical trial**

**Index**

**Additional materials:**

Methods (Page 3)

PANEL A: Criteria for division of apheresis Covid-19 Convalescent Plasma by neutralizing antibody titre and final amount of Arbitrary Units per aliquot (pag.4)

Panel B: Participant timeline: daily blood test and monitored clinical parameters to report on Case Report Forms (pag.5)

SOFA score (pag. 6)

Results (Page 7)

Figure 1s. Time to seroconversion to IgG anti-SARS-CoV-2 (A) and to virus clearance from plasma (B) and respiratory tract (C) by treatment arm (pag.8)

Figure 2s. Variation of IgG anti SARS-CoV-2 (A) and of SOFA score (B) during hospitalization, by treatment arm (pag.9)

Table 1s. Adjusted comparisons for 30 days mortality of Standard Plasma and Covid-19 Convalescent Plasma (experimental arms) with Standard of Care (Control arm) (pag.10)

Table 2s. Frequency and percentage of patients with altered laboratory values at baseline and during hospitalization (within 30 days since randomization) and 30-day mortality, by treatment arm. (pag. 11)

Table 3s. Adverse Events related and not related to plasma infusion (pag.12)

**ADDITIONAL MATERIALS**

**METHODS**

**Eligibility criteria for Centres**

Any Certified Blood transfusion Centre of Piemonte and Valle d’Aosta Regions and medical doctors, biologist, Lab technicians, nurses working in these Centres were allowed to collect, screen, prepare, inactivate, assign plasma components to specific patients anyone for his/her role. Medical doctors working in the transfusion centre were responsible for patient randomization, plasma issuing in masking way, data collection and data entry on web-based data register.

Any Public Health System Hospital of Piemonte and Valle d’Aosta Regions treating COVID-19 patients.

**Individuals who performed intervention.**

All medical doctors in the following specialization list: Anaesthetists, pneumology, infective disease, Intensive care, internal Medicine.

**Donors of COVID-19 convalescent Plasma: Inclusion Criteria**

- Voluntary non-remunerated donors
- 18 yrs <age<60 yrs if first time donor; 18 yrs<age<65 yrs if repeat donor
- Nulliparous and non-pregnant females
- Never transfused males and females
- Indirect antiglobulin test (IAT): negative
- Accomplish to all selection criteria for Italian law on blood donors
- Demonstrated previous SARS-CoV-2 infection by positivity to RNA testing by RT-PCR on nasopharyngeal swab or on bronchoalveolar lavage or presence of antibodies to SARS-CoV-2.
- Resolution of symptoms since at least 14 days
- Demonstration of infection resolution by two negative nasopharyngeal swab (at least 24 hours apart)
- Presence of anti-SARS-CoV-2 antibodies with a CLIA assay validated for neutralizing antibodies described in following paragraph
- Signed Informed consent

**Who was taking informed consent**

- Medical doctor working in transfusion centres asked to sign an informed consent to plasma collection by apheresis to COVID-19 Convalescent donors after describing procedure, discussing potential side effects and potential benefits for patients.
- Medical Specialist working in COVID-19 wards were responsible of collecting informed consent from eligible patients after explaining clinical situation, potential benefit of the plasma trial, masking and randomization meanings.
- No ancillary study was planned for this trial

**Intervention description**
Arm 1 Active comparator “Standard of Care (SC)”: the best evidence treatment recommended for COVID-19 patients by National or International guidelines from enrolment until death or discharge.

Arm 2 Experimental: SC + transfusion of 3 units of standard Plasma collected before September 2019 so before SARS-CoV-2 pandemic (SP) on day 1-3-5 after randomization.

Arm 3 Experimental: SC + transfusion of 3 units of COVID-19 Convalescent Plasma (CCP) on day 1-3-5 after randomization.

SP and CCP were submitted to pathogen inactivation with Mirasol technology (Terumo) (Riboflavin + UV).

**Assay for detection of neutralizing anti-SARS-Cov2 antibodies on donors and CCP and preparation of CCP aliquots**

Neutralizing antibodies anti-SARS-CoV-2 IgG were performed with a validated Chemiluminescent-Immuno assay: SARS-CoV-2 S1/S2 IgG (Diasorin) on LIAISON instrumentation following manufacturer instructions. The assay correlates with Plaque Reduction Neutralizing Test (PRNT): samples reacting 40AU/ml correlate with PRNT >1:80, samples reacting 80AU/ml correlate with PRNT >1:160.

Donated plasma units were then tested with the same assay. Minimum levels of antibodies was 40 AU/ml corresponding to neutralizing titres >1:80.

An apheresis plasma unit was divided in multiple aliquots in the way to have CCP units containing from 10.000 to 40.000 AU of neutralizing antibodies as shown in **Panel A**.

.

| **Panel A. Criteria for division of apheresis Covid-19 Convalescent Plasma Units by neutralizing antibody titre and final amount of Arbitrary Units (AU) per aliquot.** | | | |
| --- | --- | --- | --- |
| **APHERESIS UNIT**  **IgG anti SARS-CoV-2 AU/ml** | **Number of ALIQUOTS** | **FINAL ALIQUOT VOLUME** | **AU per ALIQUOT** |
| >250 AU/ml | 6 | 100 ml | 25.000-40.000 |
| 150-249 AU/ml | 4 | 150 ml | 22.500-37.350 |
| 75-149 AU/ml | 3 | 200 ml | 15.000-29.800 |
| 30-74 AU/ml | 2 | 300 ml | 9.000-22.200 |

**Strategies for CCP assignment**

CCP units were chosen for the patient amongst AB0 compatible units trying to use different donors and to reach a median total amount of neutralizing antibodies administered during treatment (3 units) of 70.000 AU (range 50.000-100.000). Units were administered starting with the one with the highest antibody concentration leaving the lowest concentration to the third infusion. This strategy was applied in the way to give as much as antibody as possible soon in case of reactions compromising treatment or fatalities.

The mean antibody level of collected CCP was 160 AU/ml (range 30 AU/ml - >400 AU/ml) corresponding to a mean PRNT>1:320. The mean volume of produced CCP aliquots was 216 ml, with a mean content of 29000 AU per aliquot

**RT-PCR Assays on donors and patient’s plasma samples.**

An assay for detection of SARS-CoV-2-RNA on plasma samples of donors at time of plasmapheresis and of patients at day 0,2,4,6,10,21,28 and at discharge was performed on plasma samples frozen within 2 hours after collection.

To perform SARS-CoV-2 RNA quantitation, extraction was carried out on 500 µL of plasma with the fully automated instrument QIAsymphony® SP/AS, using the kit “QIAsymphony® DSP Virus / Pathogen Midi kit” and following the manufacturer instructions (extraction protocol “Virus Cell free 500_V3_DSP_default IC”. The extracted RNA was then eluted in 110 µL (Qiagen, Milan, Italy). Nucleic acid amplification, Allplex^TM^ SARS-CoV-2 Assay (Seegene, Genova, Italy) was carried out with a multiplex real-time PCR test that allows for the detection of four target genes (i.e., N, S, RdRP, E) using CFX96™ Dx (Bio-Rad Laboratories, Segrate, Italy). For quantification a Seegene high multiplex target detection & quantification technology MuDT^TM^ that enables analysis of multiple Ct (cycle threeshold) values was used.

To perform pre-screening of viral variants, extraction and amplification were done as above described, using the Allplex^TM^ SARS-CoV-2 Variants Assay (Seegene). This assay differentiates the new variants VOC-202012/01 (lineage B.1.1.7; type of spike protein mutation HV 69-70 deletion and N501Y; U.K. variant), 501Y.V2 (B.1.351; K417N, E484K and N501Y; South Africa), and 501Y.V3 (P.1; K417T, E484K and N501Y; Brazil and Japan) by multiplexing the three different mutations in S gene.

**Data collection and management: plans for assessment and collection of outcomes**

Care providers were responsible for performing blood test as scheduled for trial. A schematic diagram for blood test and measurement required in the trial is presented in **Panel B** and was included in the case report form (CRF). Care providers had to fill daily the CRF (identified with EPICLIN personal ID number) with all blood test results and measurement of the previous day and to send a copy to the blood Center. A clinician in every blood center was responsible to check that all data were complete and to insert data on EPICLIN, the web-based database. An online training for care providers and for clinicians working in the blood centers was performed before starting recruitment.

**Panel B. Participant timeline: daily blood test and monitored clinical parameters to report on Case Report Forms.**


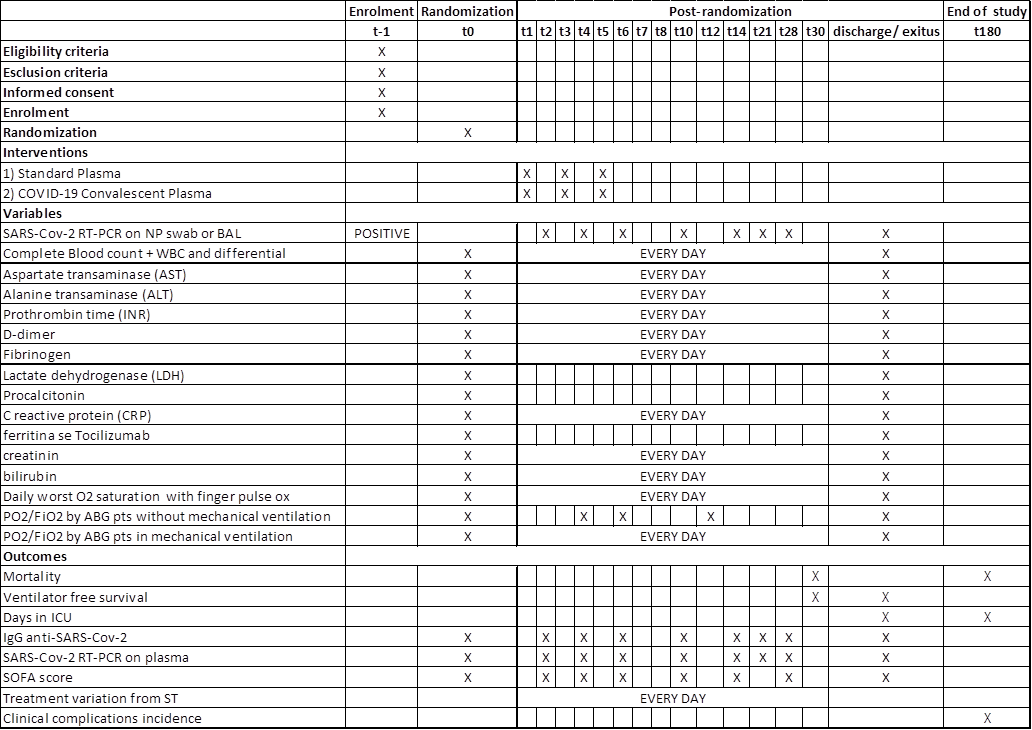
t-1: time -1: enrolment day, the day before randomization.

t-0: randomization day

t1-180: days after randomization

**SOFA SCORE**

### **Respiratory system**

| **PaO_2_/FiO_2_ [mmHg (kPa)]** | **SOFA score** |
| --- | --- |
| ≥ 400 (53.3) | 0 |
| < 400 (53.3) | +1 |
| < 300 (40) | +2 |
| < 200 (26.7) **and** mechanically ventilated | +3 |
| < 100 (13.3) **and** mechanically ventilated | +4 |

**Nervous system**

| **Glasgow coma scale** | **SOFA score** |
| --- | --- |
| 15 | 0 |
| 13–14 | +1 |
| 10–12 | +2 |
| 6–9 | +3 |
| < 6 | +4 |

**Cardiovascular system**

| **Mean arterial pressure OR administration of vasopressors required** | **SOFA score** |
| --- | --- |
| MAP ≥ 70 mmHg | 0 |
| MAP < 70 mmHg | +1 |
| dopamine ≤ 5 μg/kg/min or dobutamine (any dose) | +2 |
| dopamine > 5 μg/kg/min OR epinephrine ≤ 0.1 μg/kg/min OR norepinephrine ≤ 0.1 μg/kg/min | +3 |
| dopamine > 15 μg/kg/min OR epinephrine > 0.1 μg/kg/min OR norepinephrine > 0.1 μg/kg/min | +4 |

**Liver**[

| **Bilirubin (mg/dl) [μmol/L]** | **SOFA score** |
| --- | --- |
| < 1.2 [< 20.53] | 0 |
| 1.2–1.9 [20-32] | +1 |
| 2.0–5.9 [33-101] | +2 |
| 6.0–11.9 [102-204] | +3 |
| > 12.0 [> 204] | +4 |

**Coagulation**

| **Platelets×10^3^/μl** | **SOFA score** |
| --- | --- |
| ≥ 150 | 0 |
| < 150 | +1 |
| < 100 | +2 |
| < 50 | +3 |
| < 20 | +4 |

**Kidneys**

| **Creatinine (mg/dl)** | **SOFA score** |
| --- | --- |
| < 1.2 | 0 |
| 1.2–1.9 | +1 |
| 2.0–3.4 | +2 |
| 3.5–4.9 | +3 |
| > 5.0 | +4 |

**RESULTS**

**Characteristics of CCP administered to patients**

The mean administered amount of antibodies was 93431 AU per patient (range 56000- 100000) with the lower in a patients that didn’t complete the treatment because withdrew the consent after a mild allergic reaction. The mean amount of administered antibodies compare with that estimated to be in 3 units of 350ml CCP with a PRNT >1:160.

For patients with a body weight higher than 90 Kg a double dose was permitted if no risk of transfusion associated circulatory overload was assessed and strategy was discussed between clinicians and transfusion experts. Five patients did not complete CCP treatment, 1 because of withdrew consent immediately after randomization (no CCP transfused) while 4 transfused 2 units each (3 deaths and 1 withdrew consent after second infusion). 46 patients infused 3 CCP units, 2 patients 4 units, 1 patient 5 units and 6 patients 6 units each. A mean amount of 1130 AU of antibody per Kg were administered to patients (range 600 AU per Kg – 1800 AU per Kg).

**Adverse events**

AEs to plasma infusion were 2 cutaneous allergic reactions to CCP infusions (2 out of 195 transfused units 1,02%) and 2 cutaneous allergic reactions to SP (2 out of 179 transfused units 1,11%), one of which associated with desaturation and hypotension that needed adrenaline support.

AEs registered through follow-up were 3 pulmonary thromboembolism, 1 massive cerebral hemorrhage during ECMO, 1 myocardial infarction, 1 iatrogenic pneumothorax and 41 deaths.

Two cases of thromboembolism happened at Day 0, before any plasma infusion, and in one case at day 10, 5 days after last infusion. The myocardial infarction was described in an ischemic cardiopathic patient with a severe refractory hypoxia and severely anemic on Day 4 between second and third infusion. Causes of death were respiratory failure in all cases, associated with multi organ failure in 11 cases, with kidney failure in 2 cases, with sepsis in 2 cases, with cerebral oedema in 1 case and with massive cerebral hemorrhage in 1 case.

Numbers of AEs per randomization arm are presented in Table 1s.

| **A**   |
| --- |
| **B**   |
| **C**   |
| **Figure 1s. Time to seroconversion to IgG anti-SARS-CoV-2 (A) and to virus clearance from plasma (B) and respiratory tract (C) by treatment arm.** |

RT-PCR: Reverse Transcriptase - Polymerase Chain Reaction

| A   |
| --- |
| B   |
| **Figure 2s. Variation of IgG anti SARS-CoV-2 (A) and of SOFA score (B) during hospitalization, by treatment arm.** |

SOFA score: Sequential Organ Failure Assessment: SOFA values ranged from 0 to 24.

| **Table 1s. Adjusted comparisons for 30 days mortality of Standard Plasma and Covid-19 Convalescent Plasma (experimental arms) with Standard of Care (Control arm)** | | | | | |
| --- | --- | --- | --- | --- | --- |
|  | **Risk Ratio** | **95% CI** | | | **p** |
| Treatment arm |  |  | | |  |
| Standard Plasma *vs* control | 1.18 | 0.59 | - | 2.36 | 0.643 |
| Covid-19 Convalescent Plasma *vs* control | 1.25 | 0.61 | - | 2.57 | 0.536 |
| Severity of respiratory failure |  |  |  |  |  |
| Mild *vs* severe | 0.29 | 0.08 | - | 1.09 | 0.067 |
| Intermediate *vs* severe | 0.40 | 0.21 | - | 0.75 | 0.004 |
| Sex |  |  |  |  |  |
| Female *vs* Male | 0.74 | 0.37 | - | 1.48 | 0.395 |
| Age class |  |  |  |  |  |
| 65-74 *vs* <65 | 1.87 | 0.89 | - | 3.93 | 0.100 |
| >75 *vs* <65 | 2.17 | 0.94 | - | 4.97 | 0.068 |
| Body Mass Index |  |  |  |  |  |
| 25-29 *vs* <25 | 1.01 | 0.54 | - | 1.91 | 0.973 |
| 30+ *vs* <25 | 0.50 | 0.19 | - | 1.31 | 0.157 |
| Charlson Comorbidity Index |  |  |  |  |  |
| 1 *vs* 0 | 1.45 | 0.69 | - | 3.04 | 0.327 |
| 2+ *vs* 0 | 2.33 | 1.21 | - | 4.48 | 0.011 |
| Blood Group |  |  |  |  |  |
| A *vs* others | 0.78 | 0.42 | - | 1.43 | 0.416 |

| **Table 2s. Frequency and percentage of patients with altered laboratory values at baseline and during hospitalization (within 30 days since randomization) and 30-day mortality, by treatment arm.** | | | | | | | | | | | | |
| --- | --- | --- | --- | --- | --- | --- | --- | --- | --- | --- | --- | --- |
| **Laboratory variables** | **Standard of care (N=60)** | | | **Standard Plasma (N=60)** | | | **Covid-19 Convalescent Plasma (N=60)** | | | **Total (N=180)** | | |
|  | **Total** | **30-day mortality** | | **Total** | **30-day mortality** | | **Total** | **30-day mortality** | | **Total** | **30-day mortality** | |
| **D-Dimer (baseline)** | no. | no. | % | no. | no. | % | no. | no. | % | no. | no. | % |
| <=560 ng/mL | 7 | 0 | 0 | 6 | 0 | 0 | 10 | 1 | 10 | 23 | 1 | 4.3 |
| >560 ng/mL | 51 | 12 | 23.5 | 52 | 14 | 26.9 | 48 | 13 | 27.1 | 151 | 39 | 25.8 |
| Missing | 2 | 0 | 0 | 2 | 1 | 50 | 2 | 0 | 0 | 6 | 1 | 16.7 |
| **D-Dimer (within 30 days)** |  |  |  |  |  |  |  |  |  |  |  |  |
| <=560 ng/mL | 1 | 0 | 0 | 1 | 0 | 0 | 1 | 0 | 0 | 3 | 0 | 0 |
| >560 ng/mL | 58 | 12 | 20.7 | 59 | 15 | 25.4 | 59 | 14 | 23.7 | 176 | 41 | 23.3 |
| Missing | 1 | 0 | 0 | 0 | 0 | 0 | 0 | 0 | 0 | 1 | 0 | 0 |
| **Ferritin (baseline)** |  |  |  |  |  |  |  |  |  |  |  |  |
| <=150 ng/mL | 1 | 0 | 0 | 2 | 1 | 50 | 1 | 0 | 0 | 4 | 1 | 25 |
| >150 ng/mL | 20 | 1 | 5 | 21 | 5 | 23.8 | 19 | 5 | 26.3 | 60 | 11 | 18.3 |
| Missing | 39 | 11 | 28.2 | 37 | 9 | 24.3 | 40 | 9 | 22.5 | 116 | 29 | 25 |
| **Ferritin (within 30 days)** |  |  |  |  |  |  |  |  |  |  |  |  |
| <=150 ng/mL | 3 | 0 | 0 | 2 | 1 | 50 | 2 | 0 | 0 | 7 | 1 | 14.3 |
| >150 ng/mL | 40 | 5 | 12.5 | 36 | 8 | 22.2 | 38 | 11 | 28.9 | 114 | 24 | 21.1 |
| Missing | 17 | 7 | 41.2 | 22 | 6 | 27.3 | 20 | 3 | 15 | 59 | 16 | 27.1 |
| **CRP (baseline)** |  |  |  |  |  |  |  |  |  |  |  |  |
| <= 5 mg/L | 4 | 0 | 0 | 6 | 0 | 0 | 8 | 1 | 12.5 | 18 | 1 | 5.6 |
| > 5 mg/L | 54 | 12 | 22.2 | 53 | 15 | 28.3 | 50 | 13 | 26 | 157 | 40 | 25.5 |
| Missing | 2 | 0 | 0 | 1 | 0 | 0 | 2 | 0 | 0 | 5 | 0 | 0 |
| **CPR (within 30 days)** |  |  |  |  |  |  |  |  |  |  |  |  |
| <= 5 mg/L | 4 | 0 | 0 | 4 | 0 | 0 | 3 | 0 | 0 | 11 | 0 | 0 |
| > 5 mg/L | 55 | 12 | 21.8 | 56 | 15 | 26.8 | 57 | 14 | 24.6 | 168 | 41 | 24.4 |
| Missing | 1 | 0 | 0 | 0 | 0 | 0 | 0 | 0 | 0 | 1 | 0 | 0 |
| **LDH (baseline)** |  |  |  |  |  |  |  |  |  |  |  |  |
| <=450 UI/L | 16 | 2 | 12.5 | 16 | 2 | 12.5 | 10 | 1 | 10 | 42 | 5 | 11.9 |
| >450 UI/L | 34 | 9 | 26.5 | 36 | 12 | 33.3 | 44 | 12 | 27.3 | 114 | 33 | 28.9 |
| Missing | 10 | 1 | 10 | 8 | 1 | 12.5 | 6 | 1 | 16.7 | 24 | 3 | 12.5 |
| **LDH (within 30 days)** |  |  |  |  |  |  |  |  |  |  |  |  |
| <=450 UI/L | 18 | 2 | 11.1 | 16 | 3 | 18.8 | 10 | 0 | 0 | 44 | 5 | 11.4 |
| >450 UI/L | 41 | 10 | 24.4 | 43 | 12 | 27.9 | 50 | 14 | 28 | 134 | 36 | 26.9 |
| Missing | 1 | 0 | 0 | 1 | 0 | 0 | 0 | 0 | 0 | 2 | 0 | 0 |
| **Procalcitonin (baseline)** |  |  |  |  |  |  |  |  |  |  |  |  |
| <=2 | 45 | 9 | 20 | 51 | 12 | 23.5 | 52 | 13 | 25 | 148 | 34 | 23 |
| >2 | 5 | 3 | 60 | 5 | 3 | 60 | 1 | 0 | 0 | 11 | 6 | 54.5 |
| Missing | 10 | 0 | 0 | 4 | 0 | 0 | 7 | 1 | 14.3 | 21 | 1 | 4.8 |
| **Procalcitonin (within 30 days)** |  |  |  |  |  |  |  |  |  |  |  |  |
| <=2 ng/m | 45 | 5 | 11.1 | 46 | 9 | 19.6 | 55 | 11 | 20 | 146 | 25 | 17.1 |
| >2 ng/m | 13 | 7 | 53.8 | 14 | 6 | 42.9 | 5 | 3 | 60 | 32 | 16 | 50 |
| Missing | 2 | 0 | 0 | 0 | 0 | 0 | 0 | 0 | 0 | 2 | 0 | 0 |
| **Total** | 60 | 12 | 20 | 60 | 15 | 25 | 60 | 14 | 23.3 | 180 | 41 | 22.8 |

| **Table 3s. Adverse Events related and not related to plasma infusion.** | | | | |
| --- | --- | --- | --- | --- |
|  | **Control arm** | **Experimental arms** | |  |
|  | **Standard of care** | **Standard Plasma** | **Covid-19 Convalescent Plasma** | **Total** |
| **Adverse events related to plasma infusion, total** |  | 2 | 2 | 4 |
| mild allergic |  | 1 | 2 | 3 |
| severe allergic + desaturation and hypotension |  | 1 |  | 1 |
|  |  |  |  |  |
| **Adverse events (no plasma related), total** | 13 | 15 | 15 | 43 |
| Deaths | 12 | 15 | 14 | 41 |
| Thromboembolism | 1 | 1 | 1 | 3 |
| Myocardial infarction |  |  | 1 | 1 |
| Massive cerebral hemorrage in ECMO |  |  | 1 | 1 |
| Iatrogenic pneumothorax |  | 1 |  | 1 |
| ECMO: Extra Corporeal Membrane Oxygenation | | | | |
